# Diminished Activation of Motor Working-Memory Networks in Parkinson's Disease

**DOI:** 10.1371/journal.pone.0061786

**Published:** 2013-04-19

**Authors:** Claudia Rottschy, Alexandra Kleiman, Imis Dogan, Robert Langner, Shahram Mirzazade, Martin Kronenbuerger, Cornelius Werner, N. Jon Shah, Jörg B. Schulz, Simon B. Eickhoff, Kathrin Reetz

**Affiliations:** 1 Department of Neurology, RWTH Aachen University, Aachen, Germany; 2 Institute of Neuroscience and Medicine (INM-1, INM-4), Research Center Jülich GmbH, Jülich, Germany; 3 Jülich Aachen Research Alliance (JARA) – Translational Brain Medicine, Aachen, Germany; 4 Research Imaging Institute, University of Texas Health Science Center, San Antonio, Texas, United States of America; 5 Institute of Clinical Neuroscience and Medical Psychology, Heinrich Heine University Duesseldorf, Duesseldorf, Germany; 6 Department of Psychiatry, Psychotherapy and Psychosomatics, RWTH Aachen University, Aachen, Germany; University Medical Center Groningen UMCG, Netherlands

## Abstract

Parkinson's disease (PD) is characterized by typical extrapyramidal motor features and increasingly recognized non-motor symptoms such as working memory (WM) deficits. Using functional magnetic resonance imaging (fMRI), we investigated differences in neuronal activation during a motor WM task in 23 non-demented PD patients and 23 age- and gender-matched healthy controls. Participants had to memorize and retype variably long visuo-spatial stimulus sequences after short or long delays (immediate or delayed serial recall). PD patients showed deficient WM performance compared to controls, which was accompanied by reduced encoding-related activation in WM-related regions. Mirroring slower motor initiation and execution, reduced activation in motor structures such as the basal ganglia and superior parietal cortex was detected for both immediate and delayed recall. Increased activation in limbic, parietal and cerebellar regions was found during delayed recall only. Increased load-related activation for delayed recall was found in the posterior midline and the cerebellum. Overall, our results demonstrate that impairment of WM in PD is primarily associated with a widespread reduction of task-relevant activation, whereas additional parietal, limbic and cerebellar regions become more activated relative to matched controls. While the reduced WM-related activity mirrors the deficient WM performance, the additional recruitment may point to either dysfunctional compensatory strategies or detrimental crosstalk from “default-mode” regions, contributing to the observed impairment.

## Introduction

Parkinson's disease (PD) has traditionally been recognized as a motor disorder, characterized by bradykinesia, tremor, rigidity and postural instability. Recent research, however, revealed a more complex picture of a multicentric neurodegeneration [1], [2], where non-motor symptoms such as neuro-psychiatric, autonomic, sensory, and sleep disturbances have a profound impact on patients' morbidity and quality of life [3]. Some non-motor features such as the REM-sleep behavior disorder (RBD), depression or hyposmia may even precede the motor symptoms by many years [4]. Cognitive impairment is one of the most common non-motor symptoms in PD. It has already been observed in initial disease stages and tends to worsen over time, developing into dementia in between up to 90% of PD cases [5], [6]. Even non-demented or *de-novo* PD patients may have deficits in executive functions such as planning, concept formation, rule use, and working memory (WM) [7], [8] similar to patients with frontal lobe lesions [9]. WM impairment, however, has been argued to be one of the most relevant cognitive deficits [10], [11]. In line with the role of dopamine in WM [12], [13], several studies suggested a link between fronto-striatal dopamine deficiency and cognitive impairment in PD [14], [15]. Given that WM is not a mental capacity [16]–[20], however, it is not surprising that WM impairments in PD are not uniform. There is evidence that visuo-spatial WM is predominantly affected even in medicated PD patients [15]–[17], [19]–[23] with the most specific impairment seen in the transformation of spatial WM information into action, i.e., “memory–motor transformations” [24]–[26] with increased load or retention time leading to further performance deterioration [25], [27].

Physiologically, motor sequence reproduction involves: (1) an internal representation of the sequence, (2) WM processes to maintain this representation, and (3) the transformation of acquired representations into sequences of motor commands. While there is a large body of work [24], [26], [28]–[34] on the neuronal correlates of motor sequence learning and more abstract/sensory WM processes (such as the n-back or Sternberg task) in PD, the neurobiological underpinnings of impaired memory–motor transformations are less well understood. In this context, it is interesting to note that during sequence-learning PD patients seem to recruit additional brain regions, which was interpreted as compensation for functionally impaired pathways in order to maintain a normal level of performance [28], [35], [36]. Whether this also holds true in the context of memory–motor transformations, in which pronounced deficits seem prevalent in PD, however, remains open. The current study thus investigated the neural basis underlying motor WM in PD using functional magnetic resonance imaging (fMRI). To probe memory–motor transformations, we implemented a sequence reproduction task in which a visuo-spatial sequence was followed either by a short or long retention interval and finally a cued manual reproduction [37]. The specific aims were to investigate (i) whether memory–motor transformations and hence motor WM performance is impaired in non-demented PD patients, (ii) whether PD patients show hyperactivation similar to those interpreted as compensatory networks in sequence learning and (iii) how these behavioral and neuronal effects are modulated by recall delay and WM load.

## Methods

### Participants

23 PD patients (mean age: 67.2±6.2 (SD), male: 14) and 23 age- and gender-matched healthy control (HC) subjects (mean age: 65±4.41 (SD), male: 13) were included into this study (Table 1). All patients fulfilled the standard UK Brain Bank criteria for PD [38]. The following inclusion criteria were employed: (a) no past history of psychiatric or neurological illness including dementia and mild cognitive impairment; (b) a score of at least 26 (out of 30) on the Mini Mental Status Examination (MMSE) (c) no prior exposure to neuroleptic or antidepressant agents (d) no history of substance abuse; (e) no past medical history of severe hypertension, cardiovascular disease, autoimmune disease, or diabetes mellitus; and f) no contraindications to MRI. Additionally, we collected data of the Montreal Cognitive Assessment Test (MOCA) [39] of 12 patients (mean [SD] 26.75±1.22) and of the Parkinson Neuropsychometric Dementia Assessment (PANDA) [40] for 18 patients (mean [SD] 25.37±4.25), also revealing no signs of dementia. Importantly, none of the patients presented with impairment in activities of daily living as assessed by a detailed anamnesis. Patients were not asked to withdraw their medication; therefore, all examinations were performed in the “on-state” (levodopa equivalent daily dose (LEDD) mean: 426.15±417.45 (SD) mg).

**Table 1:** Demographic and clinical data.

	PD	Controls
**N/Gender (male)**	23/14	23/13
**Age (years)**	67.2±6.2	65±4.4
**Education (years)**	13±3	14.9±3.9
**Disease duration (years)**	4.7±4.2	n.a.
**UPDRS-III**	23.9±16.1	n.a.
**Hoehn & Yahr**	1.5±0.9	n.a.
**PDQ-39**	19.6±12.2	n.a.
**LEDD (mg)**	426.15±417.45	n.a.
**MMSE**	28.6±1.2	29.0±1.1
**Digit Span Forward (raw score)**	9±2.2	10.7±1.8
**Digit Span Backward (raw score)**	6.2±2.7	6.9±1.8
**Digit Span (standard score)**	10.6±3.2	12.2±2.2
**TMT-A (s)**	39.8±25.6	26.1±9
**TMT-B (s)**	88.9±53.7	56±20.9

Abbr.: PD, Parkinson's Disease; HC, Healthy Controls; SD, Standard Deviation; UPRDS, Unified Parkinson's Disease Rating Scale; PDQ, Parkinson's Disease Questionnaire; LEDD, Levodopa Equivalent Daily Dose; MMSE, Mini-Mental State Examination; TMT-A/B, Trail Making Test versions A and B; s, seconds; %, percent.

Before MRI scanning, all subjects underwent a clinical examination including the Unified Parkinson's Disease Rating Scale (UPDRS) [41], Hoehn and Yahr staging [42], Parkinson's Disease Questionnaire (PDQ-39) for quality of life [43], the Structured Clinical Interview for DSM-IV (SCID) to confirm absence of psychiatric comorbidity [44] and a neuropsychological test battery. The latter included the forward and backward digit span subtest of the Wechsler Memory Scale (WMS/WAIS) [45], the Trail Making Test versions A and B (TMT-A and TMT-B) [46] [47] as well as a 10 s finger-tapping test (performed three times on each side and averaged to reflect basic motor speed) and a pointing test (horizontal pointing with the index finger between two spots 30 cm apart; average time of three trials per side). All subjects were classified as right-handed by the Edinburgh inventory [48].

#### Ethics statement

Written informed consent was obtained from all participants prior to examination. The study had been approved by the local ethics committee of the RWTH Aachen University Hospital.

### MR Imaging

#### Motor working-memory task

In the motor WM task performed in the scanner, subjects had to memorize and retype (on a response key pad) a visually presented spatial sequence. At the start of each event, a visual cue (the German word “Achtung”) was displayed for 500 ms, indicating the beginning of the next trial. The cue was followed by the target stimuli consisting of red dots displayed in a sequential order on a two-dimensional schematic drawing of a hand. Each trial probed either the left or right hand and involved the indication of four (stimulus duration: 2.9 s) or five (stimulus duration: 3.5 s) randomly chosen locations corresponding the sequence to be memorized. Following a delay interval of either 500 or 7000 ms a go-cue (green circle, presented for 500 ms), instructed the participants to reproduce the sequence manually by typing the corresponding fingers on the keypad. Each of the ensuing eight different conditions (left or right hand, memory load of four or five items, delay of 500 or 7000 ms) was presented six times each. The ensuing 48 events followed in a randomized order and were separated from each other by a jittered delay between 4500 and 6500 ms. Stimuli were presented with MR-compatible goggles using Presentation® software (Neurobehavioral Systems, Inc.), and responses were collected using MRI-compatible keypads (LUMItouch, Photon Control Inc.). All subjects were familiarized with the task before scanning.

#### MRI Acquisition and preprocessing

MRI was carried out on a Siemens 3T Trio Tim scanner (Siemens Medical Solutions, Erlangen, Germany) using a gradient echo-planar imaging (EPI) sequence (TR = 2200 ms, TE = 30 ms, flip angle = 90°, matrix = 64×64 voxels, slice thickness 3 mm, field of view = 1200×1200 mm^2^). Additionally, high-resolution T1-weighted whole-brain images were acquired using an MPRAGE sequence (TR = 1900 ms, TE = 2.5 ms, matrix size = 256×256, 176 sagittal slices, voxel size = 1×1×1 mm^3^, field of view = 250×250 mm^2^).

To allow for magnetic-field saturation, image acquisition was preceded by three dummy images which were discarded prior to data analysis. Images were analyzed using SPM8 (www.fil.ion.ucl.ac.uk/spm). The EPI images were corrected for head movement by affine registration using a two-pass procedure. This included an initial realignment of all images to the first image and a subsequent realignment to the mean of the realigned images. After realignment, the mean EPI image of each participant was spatially normalized to the MNI (Montreal Neurological Institute) reference space using the unified segmentation approach [49]. The resulting parameters that define the deformation field necessary to move the participant's data into the space of the MNI tissue probability maps were then combined with the deformation field transforming between the latter and the MNI single subject template. The ensuing deformation was subsequently applied to the individual EPI volumes that were thereby transformed into the MNI single subject space and resampled at 1.5×1.5×1.5 mm3 voxel size. Finally, these normalized images were spatially smoothed with a Gaussian kernel of 8-mm full width at half-maximum.

### Data analysis

#### Behavioral data analysis

Task accuracy and response times were analyzed using the SPSS software package (SPSS v17.0, Chicago, Illinois, USA). The rate of correct reproductions, initial reaction time (i.e. the time interval between go-signal and first button press), and mean interresponse time (i.e. the time interval between the first and last button press divided by the number of items in the sequence minus one [as there are, e.g., three intervals between four responses]) was calculated for each subject and compared between conditions and groups. The effect of the between-subject factor group, and the within-subject factors delay (immediate or delayed) and memory load (4 or 5 items) on each performance measure was examined by a 2×2×2 mixed design analyses of variance (ANOVA). P-values below 0.05 were considered significant. For significant factors or interactions, pair-wise comparisons were computed with the Bonferroni correction for multiple comparisons.

#### Functional MRI data

Imaging data were analyzed using the general linear model as implemented in SPM8. In particular, we used six condition regressors reflecting encoding, immediate (direct) and delayed recall (retrieval) for the left and right hand, respectively. In addition, a parametric modulator for each regressor was introduced to capture load-related differences in local activation. In contrasts to the alternative procedure of modelling low and high load trials separately, this approach has the advantage that it allows for a more robust estimation of the main effects (based on more trials) without losing sensitivity to differences between both low- and high-load trials. Given the relatively modest performance rates in each group, we did not restrict our analysis to correct trials but rather included all those trials in which subjects pressed the required number of buttons, independently of whether the sequence was correct or not. This ensured that subjects tried to perform the task while at the same time providing a sufficient number of the estimation of neuronal responses. Each of the ensuing regressors was modelled by convolving a canonical hemodynamic response form with a boxcar reference vector reflecting the onset and duration of the respective events. That is, for the encoding, the width of the boxcar function reflected the time from the appearance of the stimulus to the end of the last item being displayed. For (immediate and delayed) recall, it corresponded from the onset of the go-cue to the last response. In addition, residual motion artefacts were modelled by including the six-parameters (three translational and three rotational) [50] estimated in the realignment preprocessing as regressors of no nuisance regressors into the model. Low-frequency signal drifts were removed by employing a highpass filter with a cut-off period of 128 seconds. After correction of the time series for dependent observations according to an autoregressive first-order correlation structure, parameter estimates of the HRF regressors were calculated for each voxel using weighted least squares to provide maximum-likelihood estimators based on the temporal autocorrelation of the data [51]. The individual first-level contrasts for each condition and its parametric modulation by load (all relative to the implicit baseline) were then fed into a second-level random-effects ANOVA. In this group analysis, mean parameter estimates were computed within in each group (controls, patients) for the three conditions (encoding, immediate recall and delayed recall) as well as their modulation by item load. The two different delays that were implemented to different delay periods represented direct and delayed retrieval. The only reason why “direct retrieval” was performed with a delay of 500 ms is to avoid attentional blink phenomena/surprise by the immediately appearing go. On the other hand the manipulation of WM load was set up to reflect easy and difficult items (low and high WM load). For that however, the available levels were rather limited as sequence length of three items or less resulted in ceiling effects in the control population (almost perfect reproduction), whereas item sequences of six or more items led to floor effects in the patient group (many patients performing at less than ten percent success). It is important to emphasize that the different magnitude ratios have no direct bearing on our analysis rather we compared no/short delay versus long delay and easy versus difficult memory load in a categorical fashion. We allowed for violations of sphericity by modeling nonindependence across images from the same subject and allowing unequal variances between conditions and subjects as implemented in SPM8.

Differences between conditions or groups were then tested by applying appropriate linear contrasts to the ANOVA parameter estimates. All effects were investigated as main effects across both respond hands, as this study was neither aimed nor well suited (given the relatively low number of trials) to study lateralization effects. Rather, left/right trials were randomized and counter-balanced only to avoid a potential confound of stimulus- or response-side. Conjoint main effects were tested by means of a conjunction analysis using the minimum statistics approach [52]. The resulting SPM(T) maps were then thresholded at P<0.05 conducting a family-wise error (FWE) correction on the cluster-level (cluster forming threshold at voxel level P<0.001; [53]). For investigation of load-related effects, a slightly more liberal cluster-level threshold of p<0.001 (uncorrected) was employed.

#### Voxel-based morphometry (VBM)

As structural brain changes may principally confound functional MRI data, we performed voxel-based morphometry (VBM) [54] to control for gray matter differences between patients and controls in the fMRI data analysis. T1-weighted images of all subjects were processed and analysed with SPM8 and the VBM8 toolbox (http://dbm.neuro.uni-jena.de/vbm). Briefly, T1-weighted images were spatially normalized by high-dimensional warping with a standard template and segmented into gray matter (GM), white matter and cerebrospinal fluid. To correct for individual brain sizes and allow comparing the absolute amount of tissue volume [55], voxel values were multiplied (“modulated”) by the non-linear component of the Jacobian determinant derived from the spatial normalization. Finally, modulated GM images were smoothed with a Gaussian kernel of 8-mm FWHM. Using a general linear model, voxel-wise gray matter differences between patients and controls were examined using independent-sample *t*-tests and by including age as a nuisance covariate. For the statistical analysis, we employed a family-wise error (FWE) corrected threshold (on cluster level) of p<0.05.

#### Anatomical allocation

All results were anatomically labeled by reference to probabilistic cytoarchitectonic maps of the human brain using the SPM Anatomy Toolbox [56], [57]. Using a Maximum Probability Map (MPM), activations were assigned to the most probable histological area at their respective locations. Details on these cytoarchitectonic regions can be found in the following publications reporting on the cerebellum [58], thalamus [59], premotor cortex (PMC, BA 6; [60]), primary motor cortex (M1, BA 4a, BA 4p) [61], primary somatosensory cortex (BA 3a, BA 3b) [62], [63]), parietal operculum (OP4) [64], insula (lg2) [65], Broca's region (BA 45) [66], inferior, superior parietal cortex and superior parietal lobule (IPC, SPC and SPL; PGp; 7P; 7PC) [67]–[69], intraparietal sulcus (IPS; hlP1; hlP3) [70], visual cortex (BA 17; BA 18 [71]; hOC3 (V3); hOC4 (V4) [72]; hOC5 (V5/MT+)) [73] and hippocampus (Hipp (EC)) [74]. Brain regions not yet histologically mapped were macroanatomically labeled by reference to the WFU Pickatlas (version 2.4) [75].

## Results

### Clinical and neuropsychological data

Results of the clinical and neuropsychological examination are summarized in Table 1. There was no significant difference between both groups with respect to age (p = 0.38), gender (p = 0.59), years of education (p = 0.06) or MMSE score (p = 0.15). PD patients demonstrated significant deficits in nearly all neuropsychological tests as indicated by two-sample *t*-tests. In particular, they performed worse in forward digit span subtest of the WMS (*t* (44) = −2.77, p = 0.008); TMT-A (*t* (44) = 2.415, p<0.02) and TMT-B (*t* (44) = 2.73, p<0.009). Increase in completion time between the TMT-B and TMT-A, which may be interpreted as a marker for executive control, was also significantly elevated (worse) in PD patients (*t* (44) = 2.54, p<0.015). As expected, patients were also significantly slowed in the pointing and finger-tapping examinations. The only neuropsychological test not reaching statistical significance was the backward digit span subtest of the WMS (p<0.2) in which the patients recalled on average one item less than the controls but both groups showed a pronounced inter-individual variability. The WMS age-appropriate standard scores that have been converted from the sum of the raw scores of both, the digit span forward and backward tests, however demonstrated significantly more decline in PD patients than in controls (*t* (44) = −2.035, p<0.048).

### Behavioral data

Multiple mixed design ANOVAs confirmed that performance accuracy (i.e. correct sequence reproductions) was significantly lower in PD patients than in HC across all conditions [F(1, 41) = 11.329; p<0.002]. Also, higher memory load [F(1, 44) = 68.481; p<0.001] and delayed response initiation [F(1, 44) = 13.496; p<0.001] caused additional decline in performance accuracy in both groups. Neither factor, however, showed a significant interaction with “group”, indicating that patients and controls perform worse with longer sequences or delays. Likewise, there was no significant load×delay interaction. Furthermore, PD patients used more time to respond as indicated by significantly higher mean interresponse time in PD compared to HC [F(1, 44) = 4.219; p = 0.046]. Likewise, higher memory load but not delay periods caused longer interresponse time intervals in both groups [F(1, 44) = 63.481; p<0.001]. There was no significant interaction between these factors or with group. Finally, initial reaction time was prolonged by delayed response initiation compared to immediate responses [F(1, 44) = 18.161; p<0.001] but not significantly different between low- and high-load conditions. Please see also Table S1.

### Functional MRI Data

Condition-related effects were tested as main effects across all participants, i.e. both groups, and are shown in the supplementary material (Figures S1, S2, S3, S4, S5, and S6). A detailed assessment of task-related effects (against implicit baseline), differences between condition (encoding, direct and delayed recall) and load-related (higher activation in the five compared to the four item condition as reflected by the parametric modulator) is outside the scope of this work. Although we are not able to eliminate a potential limitation of the current study, which might be a possible confounding effect of motor execution during the task, we would nevertheless like to note, that all effects resonate well with known networks for working memory and memory–motor transformations (e.g. [22], [37], [76]–[79]), confirming the effectiveness of our experimental setup and the appropriateness of the imaging and analysis approach.

#### Encoding

**Figure 1:**
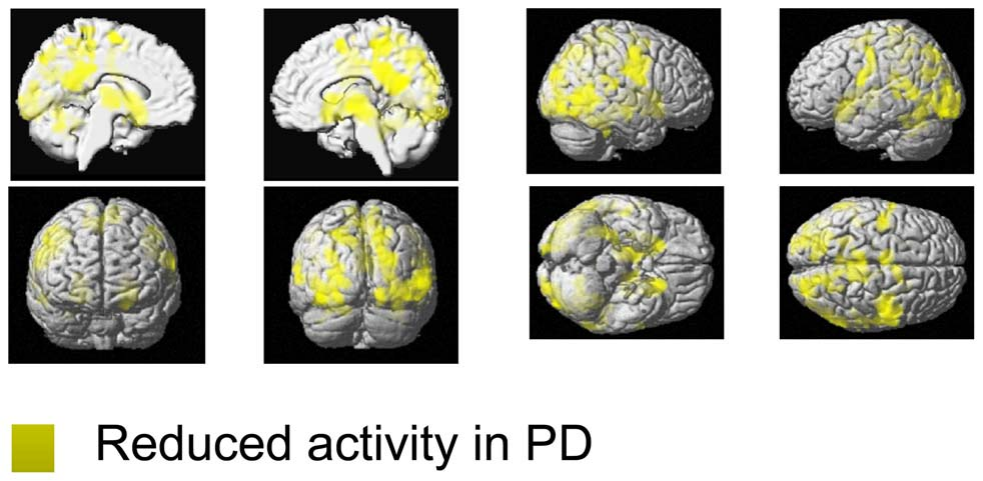
Functional working-memory related correlates in PD and controls during the encoding phase. Regions showing significantly lower activity (yellow) in PD relative to healthy controls during the encoding phase of the motor WM task. All significant effects are displayed on the MNI single subject template and the color bar represents T-values.

**Figure 2:**
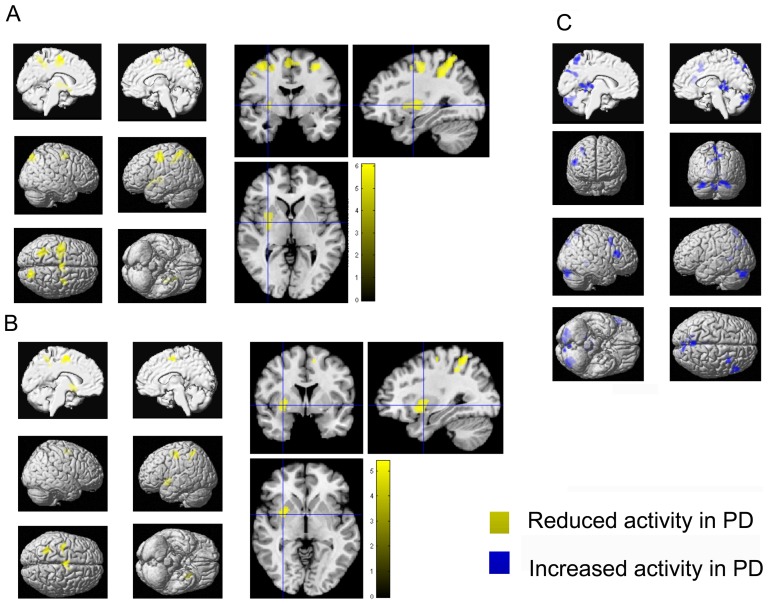
Functional working-memory related correlates in PD and controls during direct and delayed recall. **A–C**) Regions showing significantly lower activity (yellow) in PD relative to healthy controls during **A**) direct recall and **B**) delayed recall. **C**) Regions showing significantly higher activity (blue) in PD relative to healthy controls during delayed recall. All significant effects are displayed on the MNI single subject template and the color bar represents T-values.

**Figure 3:**
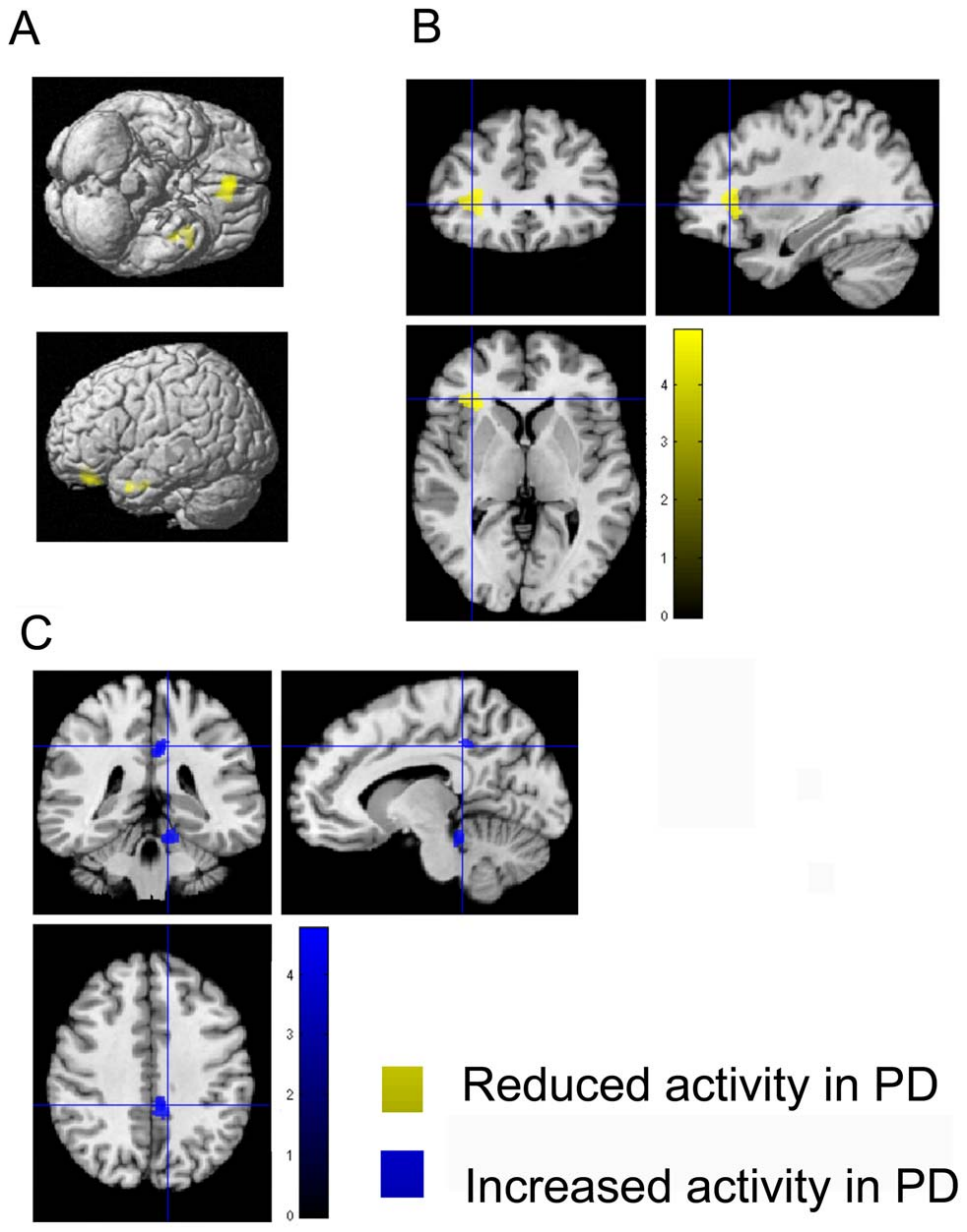
Functional working-memory related correlates in PD and controls during load-related modulation. **A–B**) Regions showing significantly lower load-related modulation in PD during the encoding phase **A**) and delayed recall **B**). **C**) Regions showing significantly higher load-related modulation in PD relative to healthy controls during delayed recall. All significant effects are displayed on the MNI single subject template and the color bar represents T-values.

**Figure 4:**
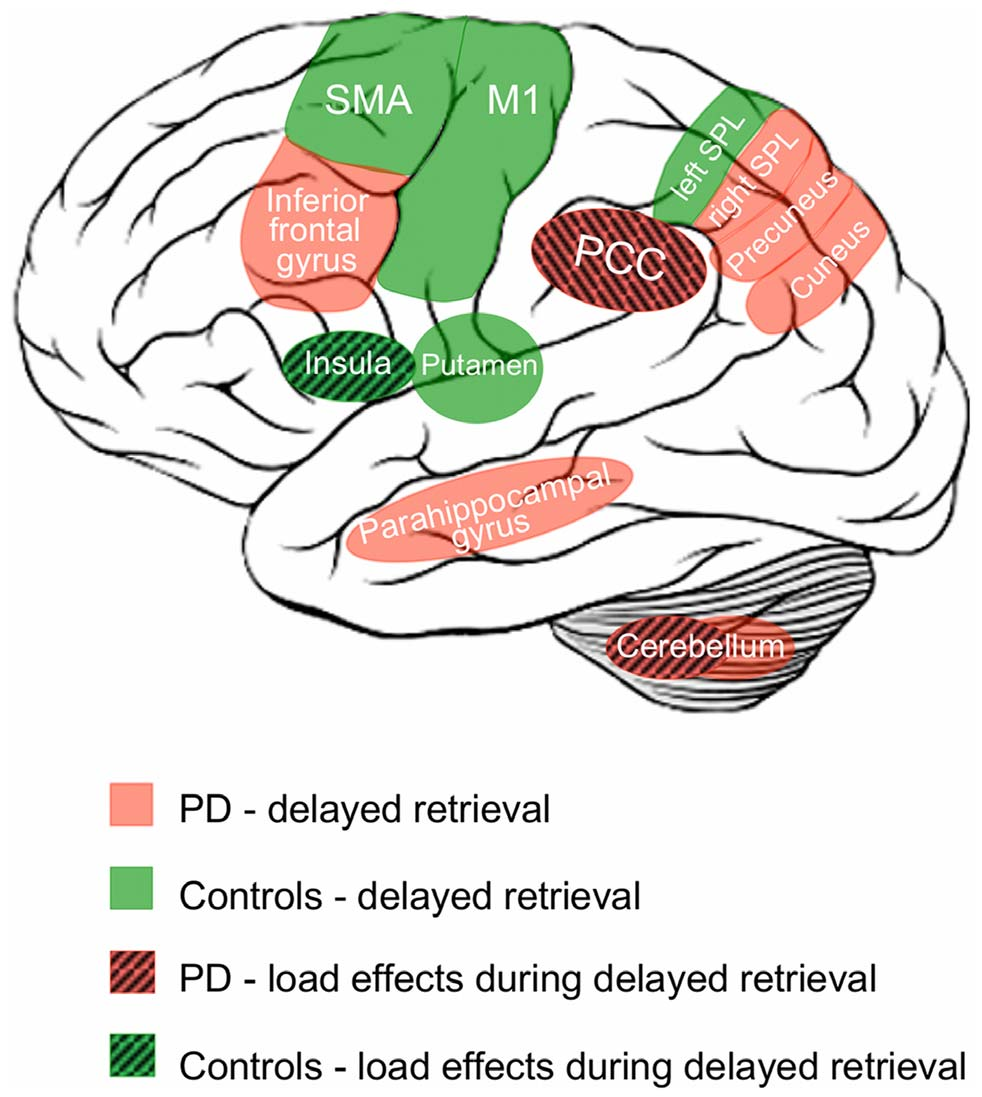
Schematic overview of working-memory related activation patterns in PD and controls. Schematic summary of brain regions showing task- or load-related differences during the delayed recall condition representing memory - motor transformation.

FMRI results are summarized in Tables 2, 3, and 4 as well as visualized in Figures 1, 2, 3, and 4. Relative to controls, PD patients showed reduced encoding-related activity in a large, bilateral network (Table 2A, Figure 1). In particular, reduced activation in patients was most pronounced in the bilateral putamen, extending to the bilateral thalamus and temporo-occipital cortex. Furthermore, the bilateral temporal gyrus, bilateral superior parietal cortex, bilateral dorsal and ventral occipital cortex including left posterior fusiform gyrus and left cerebellar lobule VI were less activated in patients. Further reductions were observed in the bilateral pre- and primary motor cortex, bilateral inferior frontal gyrus, right precuneus, medial superior parietal cortex, bilateral SMA as well as the right inferior parietal cortex. For additional information including cluster size, stereotaxic location and histological allocation confer Table 2A. We found no region that showed significantly higher activation in PD patients relative to controls during encoding (Table 3A).

**Table 2:** Reduced working memory related functional MRI results in PD compared to controls.

Macroanatomical location	Cytoarchitectonic location	MNI coordinates of local maxima			z-score	kE
		x	y	z		
**A) Reduced activation in PD compared to controls during encoding**						
Left Putamen		−24	15	−9	6.82	17869
Right Putamen		21	17	−11	5.65	
Right thalamus		12	−15	−3	6.66	
Right occipital cortex	hOC5	54	−66	3	6.65	
Right superior parietal occipital cortex		17	−65	47	6.1	
Right dorsal occipital cortex		29	−87	26	5.71	
Right ventral occipital cortex	FG1	35	−69	−12	5.57	
Right inferior temporal cortex		33	−50	−20	5.51	
Right lateral occipital cortex		60	−51	0	4.9	
Right superior temporal gyrus		62	−54	11	4.62	
Left thalamus		−12	−14	2	4.78	
Left dorsal occipital cortex		−24	−89	9	6.41	5089
Left ventral occipital cortex	FG1	−39	−86	−12	4.9	
Left inferior temporal cortex		−41	−60	−14	5.21	
Left cerebellum Lobule VI	Lobule VI	−17	−65	−27	4.48	
Left occipital cortex	hOC5	−45	−72	0	4.17	
Right precentral gyurs	Area 6	38	−8	45	6.08	4087
Right motorcortex	Area 4p	42	−11	38	6.04	
Right inferior precentral gyrus	Area 4p	54	−3	27	5.25	
Left middle occipital gyrus		−30	−69	26	5.95	876
Left superior parietal occipital cortex		−15	−78	42	4.4	1038
Precuneus		5	−54	17	5.9	6715
Posterior cingulate cortex		6	−39	26	5.14	
Retrosplenial cortex		12	−62	23	4.79	
Right paracentral gyrus	Area 3a/Area 4p	14	−33	59	4.49	
Right paracentral gyrus	Area 3a/Area 4p	−9	−35	72	4.33	
Left superior parietal lobule	Area 7PC	−24	−51	48	5.7	423
Left Motorcortex	Area 4p	−42	−14	36	5.18	1709
Left inferior frontal gyrus	Area 3a/Area 4p	−45	−9	30	4.99	
Left superior temporal gyrus		−62	−54	6	5.01	1149
Left parieto-occipital junction		−44	−38	26	4.18	
SMA	Area 6	−5	−9	65	4.91	1118
SMA	Area 6	8	3	59	4.7	
Right inferior parietal cortex	Area PFcm	62	−29	15	4.64	579
Right inferior parietal cortex	Area PFcm	51	−38	21	4.49	
Right middle temporal gyrus		56	−17	−11	4.5	355
**B) Reduced activation in PD compared to controls during direct recall**						
Left primary motor cortex	Area 4a	−42	−14	47	5.96	2930
Left SMA	Area 6	−3	−8	62	5.12	
Left dorsal precentral gyrus	Area 6	−39	−6	53	5.42	
Left superior parietal lobule	Area 7PC	−30	−53	57	5.68	1365
Left intraparietal sulcus	Areas hIP1–3	−30	−42	42	5.26	
Right superior parietal lobule	Area 7P	14	−78	54	5.84	807
Left Putamen		−30	−11	3	4.8	676
Right dorsal precentral gyrus		35	−3	51	5.33	397
**C) Reduced activation in PD compared to controls during delayed recall**						
Left Putamen		−32	3	−8	5.12	646
SMA	Area 6	−3	−8	59	5.31	590
SMA	Area 6	11	0	56	3.82	
Left superior parietal lobule	Area 7PC	−32	−50	56	5.3	547
Left primary motor cortex	Area 4	−39	−15	51	5.28	493
**D) Reduced load effects in PD compared to controls**						
**Encode**						
Right Medial Orbitofrontal cortex		2	41	−20	4.87	582
Left anterior inferior temporal sulcus		−39	−5	−29	4.52	301
**Direct recall**						
No significant effect						
**Delayed recall**						
Left anterior Insula		−30	27	3	4.87	391

Abbr.: kE: cluster size; x, y, z: MNI co-ordinates; PD, Parkinson's disease, HC healthy controls.

**Table 3:** Increased working memory related activation in PD.

Condition	Macroanatomical location	Cytoarch. location	MNI coordinates of local maxima			Z-score	kE
			x	y	z		
**A) Increased activation in PD compared to controls during encoding, recall and delayed recall**							
**Encode**	no significant effects						
**Direct recall**	no significant effects						
**Delayed recall**	Left posterior parahippocampal gyrus		−17	−51	6	4.49	1586
	Right posterior parahippocampal gyrus		20	−42	−3	4.21	
	Retrosplenial cortex		3	−38	9	4.16	
	Right cerebellum	Lobule VIIa	24	−83	−23	4.17	1194
	Left cerebellum	Lobule VIIa	−27	−72	−23	3.66	921
	Right inferior frontal gyrus	Area 45	51	26	21	4.84	566
	Right superior parietal occipital cortex		9	−84	48	4.3	480
	Right posterior middle frontal gyrus		36	12	50	4.49	427
	Left medial superior parietal lobule	Area 7A	−3	−62	66	4.97	362
**B) Increased load-effects in PD compared to controls**							
**Encode**	no significant effects						
**Direct recall**	no significant effects						
**Delayed recall**	Right cerebellum	Lobule I–IV	11	−36	−21	4.31	366
	Right posterior cingulate	Area 7A	5	−41	35	4.68	362

Abrr.: kE: cluster size; x, y, z: MNI co-ordinates; PD, Parkinson's disease, HC healthy controls.

**Table 4:** Condition by group interaction.

	Macroanamtomical location	Cytoarchitectonic location	MNI coordinates of local maxima			z-score	kE
			x	y	z		
**A) Reduced activation in PD for direct recall**							
	Right posterior superior parietal lobule	7P	14	−78	54	5.94	804
	Left posterior superior frontal gyrus		−38	−3	51	4.74	669
**B) Increased activation in delayed recall in PD**							
	Right cerebellum	Lobule VIIa Crus I	24	−83	−23	4.21	1102

#### Direct and delayed recall

During immediate recall, when subjects had to retype the memorized sequences after a delay of only 500 ms, PD patients showed reduced activation relative to controls in the left precentral gyrus, left SMA, bilateral dorsal precentral gyrus, bilateral superior parietal lobule, left intraparietal sulcus and middle and posterior parts of the left putamen (Table 2B, Figure 2A). In turn, no brain area showed significantly increased activation in PD relative to controls (Table 3A).

In the long delay condition (in which the subjects had to reproduce the sequence after 7000 ms) PD patients featured significantly less activation in the left putamen, superior parietal cortex and precentral gyrus as well as in bilateral SMA (Table 2C, Figure 2B). Additionally, PD patients showed increased bilateral activation (compared to controls) in the posterior parahippocampal gyrus and cerebellar lobule VIIa. Moreover, increased activation was found in the right inferior frontal gyrus, and the posterior midline including the retrosplenial cortex, while in the left hemisphere increased activation was found in the medial superior parietal cortex (Table 3A, Figure 2C). Again, additional details for all effects, including cluster size, stereotaxic location and histological allocation, are provided by the tables 2B/C and 3A. A schematic overview of working-memory related activation patterns in PD and controls is illustrated in Figure 4.

#### Load-related modulations

PD patients showed significantly lower load-related effects during encoding, i.e., significantly less modulation of neuronal activity when memorizing five as compared to four items in the right medial orbitofrontal cortex and the left anterior inferior temporal sulcus relative to healthy controls during encoding (Table 2D, Figure 3A). During delayed recall, PD patients showed significantly lower load-related modulation of activity in the left anterior insula (Figure 3B). In contrast, PD patients showed significantly higher load-related modulation during delayed recall in the right posterior cingulate cortex and right cerebellar lobule I–IV (Table 3B, Figure 3C). Again, details regarding details on cluster size, stereotaxic location and histological allocation are given in the tables 2D and 3B, for an overview please see Figure 4.

#### Condition by group interaction

Furthermore, we compute the “group×task” interaction to statistically assess, whether the factor “group” (PD vs. controls) modulates the within-group factor “task” (direct vs. delayed retrieval). Evidently, two possible interaction effects may be computed, representing the opposite direction of the “group×task” interaction. In particular, given the order of the relevant regressors as *ConDirect ConDelayed PatDirect PatDelayed*, these two terms are [1 −1 −1 1] and [−1 1 1 −1].

The first tests, whether the difference in the neuronal activation between controls and patients for direct retrieval is greater than the difference between the two groups for delayed retrieval (ConDirect – PatDirect)>(ConDelayed – PatDelayed). Alternatively, however, this may be interpreted as a test, where the difference in neuronal activation between patients and controls for delayed retrieval is greater than the difference between the two groups for direct retrieval (PatDelayed – ConDelayed)>(PatDirect – ConDirect). To differentiate these two alternative accounts for the (same) [1 −1 −1 1] interaction, we constrained our analysis by a conjunction with the minuend of the two alternatives, i.e., forcing the direction of the observed effect. The contrast [1 −1 −1 1] ∩ [1 0 −1 0] hence tests for regions, where patients show a specific reduction in activation during direct retrieval (ConDirect – PatDirect)>(ConDelayed – PatDelayed). Testing for this interaction at p<0.05 (cluster-level FWE correction for multiple comparisons, cf. Fig. S7a; Table 4A), yielded two significant regions in the left posterior superior frontal gyrus and right posterior superior parietal lobule (area 7P) in which activity in PD patients was specifically reduced during direct retrieval. In turn [1 −1 −1 1] ∩ [0 −1 0 1] tests for regions, where patients show a specific increase in activation during delayed retrieval (PatDelayed – ConDelayed)>(PatDirect – ConDirect). Testing for this interaction at p<0.05 (cluster-level FWW, cf. Fig. S7b; Table 4B), yielded one significant effect in the right cerebellum (lobule VIIa Crus I).

The second interaction term [−1 1 1 −1] tests, whether the difference in the neuronal activation between controls and patients for delayed retrieval is greater than the difference between the two groups for direct retrieval (ConDelayed – PatDelayed)>(ConDirect – PatDirect). Alternatively, however, this may be interpreted as a test, where the difference in neuronal activation between patients and controls for direct retrieval is greater than the difference between the two groups for delayed retrieval (PatDirect – ConDirect)>(PatDelayed – ConDelayed). Testing for this interaction yielded no significant effect, even when lowering the threshold to p<0.001 uncorrected.

### Voxel-based morphometry

In our sample of PD patients and age- and sex-matched controls, no significant differences in gray-matter volume or differences in total brain volume were detected. That is, we found no evidence for significant (at p<0.05 corrected for multiple comparisons) regionally specific (given that total brain volume was included as a covariate into the analysis) atrophy in our groups of PD patients. In other words, the examined patients showed the above described neuropsychological and functional differences in spite of neither featuring clinical signs of dementia (dementia screening tests) nor significant atrophy (VBM).

## Discussion

This fMRI study investigated aberrations in neuronal responses during a motor WM task in non-demented patients with PD. In spite of absence of clinical dementia and significant brain atrophy, we demonstrated that: (I) PD patients performed significantly worse on the motor WM task than closely matched healthy controls. II) There was no group by load or delay interaction on performance rates. (III) Impaired task performance was associated with reduced task-related activity in all phases but in particular during encoding. (IV) During sequence encoding PD patients showed reduced activity in a widespread network comprising the basal ganglia, motor, cingulate and parieto-occipital cortices. (V) During recall, reduced activation was found in cerebral motor networks, superior parietal structures, and the putamen. Increased activation was found in the bilateral posterior parahippocampal gyrus and the posterior cerebellum as well as in the posterior midline when recall was delayed. (VI) In PD, significantly reduced load-modulations were observed in the orbitofrontal cortex and anterior insula, while the posterior cingulate cortex and the cerebellum showed increased load-modulation in patients.

### Aberrant encoding-related activity in PD

The encoding phase involves stimulus processing and the formation of transient motor representations [80]. In particular, there is solid evidence for subliminal activation of the motor system, i.e. covert action, simulation being triggered by observing an action or receiving information representing actions such as words or motor-related spatial cues as in the present experiment (for review: [81]). The observed widespread reduction of activity during encoding in PD is in line with previous studies reporting reduced activation during action simulation [82], [83] and motor programming [84] in these patients. This interpretation as implicitly triggered motor activation holds particularly for the effects in premotor cortices [85] and matches previous reports of malfunctioning mesial motor areas in PD [86]–[88], i.e., regions strongly involved in the interface between cognitive and motor processes. The dorsal lateral premotor cortex, in turn, is predominantly associated with planning and execution sensory-guided movements [89] and externally triggered movements [90]. Our study thus provides evidence for reduced stimulus-driven triggering of activation within the cortical motor system by highly associative action-related spatial stimuli. Furthermore, the reduced activation in the putamen during both encoding and subsequent recall is well in accordance with earlier fMRI studies linking this region to impaired spatial motor WM [91], [92]. The putamen was shown to actively contribute to stimulus maintenance [93] and also associated with episodic memory encoding [94]. Reduced activation in the putamen may thus reflect potentially dopamine-dependent aberrations during the maintenance of motor representations. Decreased activation in the posterior parietal lobe and in particular the precuneus finally resonates well with recent findings, that this region plays a key role in multiple higher cognitive processes [95] including attentive tracking [96], visuo-spatial [97] and motor imagery [98]–[101]. It may hence represent a hub of cognitive functioning, which is disturbed in patients with PD resulting in impaired task performance. When further considering the recently discussed association of the medial superior parietal cortex with imaginative processes and prospective cognition (but not actual task execution in many goal directed [motor] tasks, cf. [102]), it may be speculated, that insufficient imagery and simulation within or controlled by the precuneus may represent a key component of this reduced task performance in PD patients.

In summary, our results thus suggest that impaired motor WM in patients with PD may represent a composite deficit related to insufficient triggering of implicit enactment by the cortical motor system, reduced basal ganglia activation resulting in impaired transfer into short term storage and finally reduced simulation and imagery under the guidance of superior and medial parietal cortices.

### Aberrant recall-related activity in PD

Delayed response initiation and prolonged interresponse times may be regarded as direct reflection of bradykinesia, a clinical hallmark of PD. Longer delay intervals furthermore decreased task performance but did not result in longer interresponse times and actually speeded up response initiation. Furthermore, there was no significant group by delay or load interaction. These results hence point to dissociation between task difficulty and motor slowing, which are both present in patients with PD but reflected in different measures derived from the employed motor WM task. The PD-related slowing is neuronally reflected by decreased activation in the (pre-) motor and (particularly superior) parietal cortex as well as the left putamen. All of these areas are directly involved in the preparation and execution of voluntary movements. Consequently, we would conjecture that their reduced activation should best be interpreted as neuronal correlates of the slowed motor response in the patients, rather than with respect to the impaired (cognitive) task performance. In other words, whereas the reduced activity during encoding may be primarily responsible for deficits in the correct encoding and hence recall of sequences, most of the effects seen during the reproduction period may be attributable to impaired motor control and difficulties in initiating and performing the sequence reproduction.

In contrast, increased activation was observed only in the context of delayed recall in several regions, including the parahippocampus. The latter findings is particularly thought-provoking given reports on PD pathology in this region [103], [104] and its involvement for spatial localization tasks [105]. Its strategic position within the medial temporal lobe makes it well suited to participate in the long-term storage [106] of currently available information [107] indicating a correspondence to the integrative functions of an episodic buffer [108] that is predictive of subsequent long-term memory [109]. In sequence learning tasks, increased parahippocampal activation [110]–[112] was found in PD subjects with better learning performance [33]. In contrast to these findings indicating a supportive role, we observed parahippocampal hyperactivity in spite of deficient task performance. This may relate to the concurrently decreased activation of cortical motor systems but potentially also to the increased activation of the posterior cingulate cortex. The latter is particularly interesting as this region is frequently associated with the default mode system of the human brain [102] and failure to deactivate it may lead to impaired task performance. While it is tempting to speculate about a dysbalance between the default mode and cortical motor network during the delayed recall of action sequences from working memory, further data seems to be first needed to dissociate motor (bradykinesia) related effects from neuronal correlates of cognitive performance and supportive from disruptive effects. We would hence only conclude that impaired task performance may result from a complex interplay of reduced (cortical and striatal motor system) and increased (parahippocampus) beneficial as well as potentially detrimental (posterior cingulate) activation.

### Effects of increased memory load

Increased memory load significantly reduced the accuracy of sequence recall in both groups without a particular effect on PD patients or an interaction with delay. Nevertheless, decreased load-related effects in PD were observed in the medial orbitofrontal and temporal cortices (during encoding) and in the anterior insula (during delayed recall). In turn, activation was increased in the posterior cingulate cortex. The latter set of effects may be particularly relevant, as these two regions are considered part of antagonistic “saliency”/task positive (anterior insula [113]) and “default”/task-negative (posterior cingulate [114]) networks. This argues for a dysbalance between these networks in PD that may result in increased cross-talk from resting-state networks, insufficient recruitment of task-relevant and attention-related areas and ultimately impaired task performance. Finally, it is important to point out, that most effects in the current study were observed when looking at delayed rather than immediate recall in spite of the fact that we observed no significant group×delay interaction, i.e., performance was not particularly impaired in this task. A potential explanation for this discrepancy is the *per se* higher difficulty of this condition (cf. lower performance across both groups) and the additional involvement of memory – motor transformations. The latter may not be necessary in the immediate recall condition, where sensory and (implicitly triggered) motor representations may still be active.

## Conclusions

Here we investigated differences in task performance and neuronal correlates in a motor WM task between non-demented PD patients and healthy control subjects. We found that reduced task performance was associated with widespread attenuation of task-related activity in a bilateral WM network. Furthermore, bradykinesia seems differentiable from cognitive performance and related to hypoactivity of the striatal and cortical motor system. Moreover, we observed increased activation in limbic areas that were previously associated with beneficial (parahippocampus) and detrimental (posterior cingulate) effects in PD patients.

## Supporting Information

Figure S1 Left side - main effect (compared to resting baseline across both groups). Right side - load related effects during encoding (main effects across both groups).
(TIF)

Figure S2 Left side - main effect of direct recall (compared to resting baseline across both groups). Right side - load related effects during direct recall (main effects across both groups).
(TIF)

Figure S3 Left side - main effect of delayed recall (compared to resting baseline across both groups). Right side - load related effects during delayed recall (main effects across both groups).
(TIF)

Figure S4 Left side – increased activation during encoding relative to direct recall across both groups. Right side - increased activation during encoding relative to delayed recall across both groups.
(TIF)

Figure S5 Left side – increased activation during direct recall relative to encoding across both groups. Right side - increased activation during delayed recall relative to encoding across both groups.
(TIF)

Figure S6 Left side – conjunction between direct and delayed recall across both groups. Right side - conjunction between encoding, direct and delayed recall across both groups.
(TIF)

Figure S7 A - Interaction (ConDirect – PatDirect)>(ConDelayed – PatDelayed): Regions in which patients showed a significant specific reduction of activity during direct retrieval as tested by the interaction (ConDirect – PatDirect)>(ConDelayed – PatDelayed) in conjunction with the respective main effect (ConDirect – PatDirect), as well as the mean parameter estimates and 90% confidence intervals for the individual conditions at the location of the local maxima. B - Interaction (PatDelayed – ConDelayed)>(PatDirect – ConDirect): Regions in which patients showed a significant specific increase of activity during delayed retrieval as tested by the interaction (PatDelayed – ConDelayed)>(PatDirect – ConDirect) in conjunction with the respective main effect (PatDelayed – ConDelayed), as well as the mean parameter estimates and 90% confidence intervals for the individual conditions at the location of the local maxima.
(TIF)

Table S1 **Working memory task performance accuracy in patients with Parkinson's disease (PD) and healthy controls (HC) during direct recall, delayed recall and all conditions.** Hits and misses are given for the 4-sequence and 5-sequence.
(DOC)
