# Supplementary figures and images for: Diminished Activation of Motor Working-Memory Networks in Parkinson's Disease
Source: PLoS One. 2013 Apr 19;8(4):e61786. doi: 10.1371/journal.pone.0061786 (PMC3631252; doi:10.1371/journal.pone.0061786)

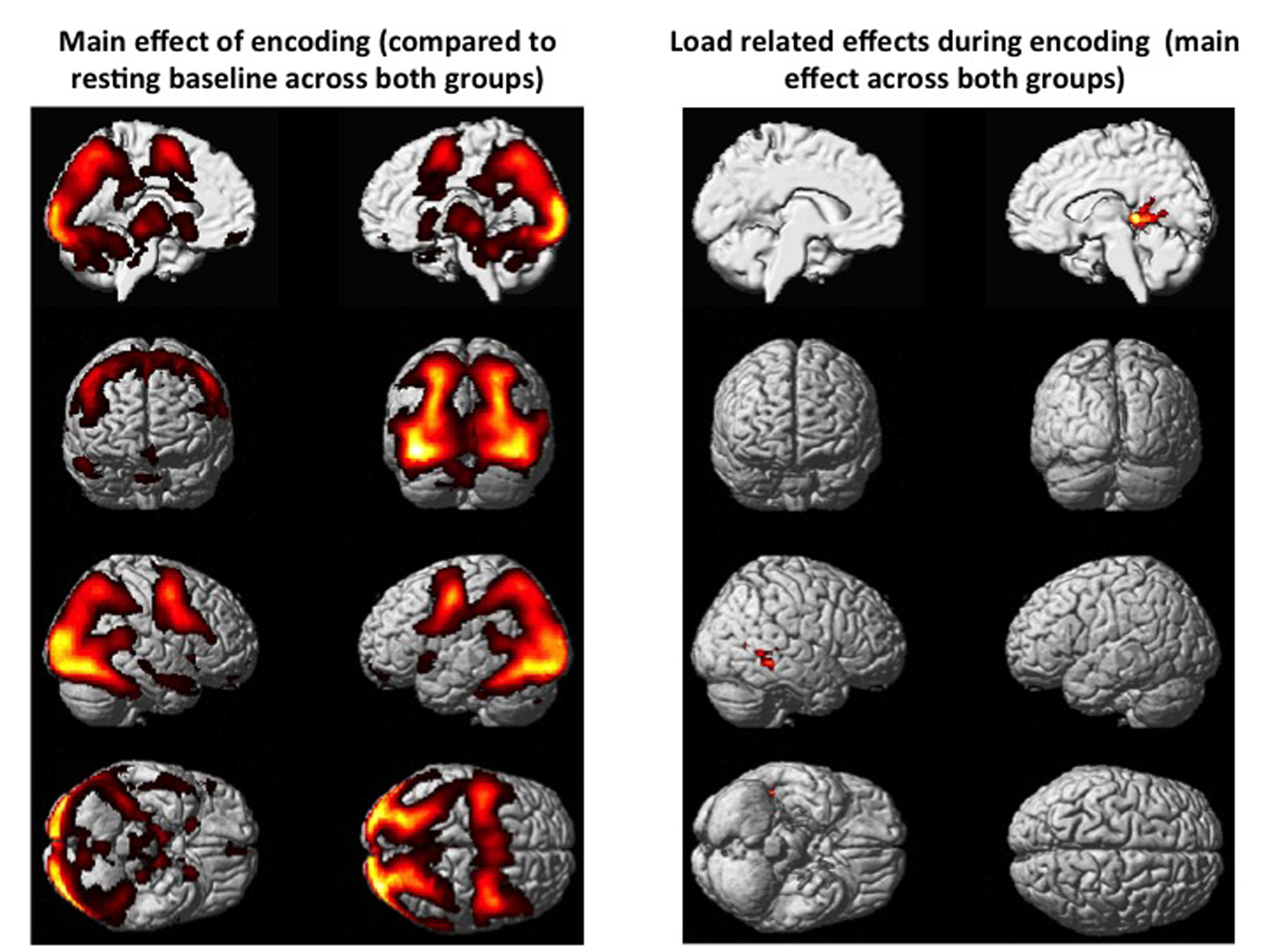

Supplement: Figure S1 — Left side - main effect (compared to resting baseline across both groups). Right side - load related effects during encoding (main effects across both groups). (TIF) [file pone.0061786.s001.tif]

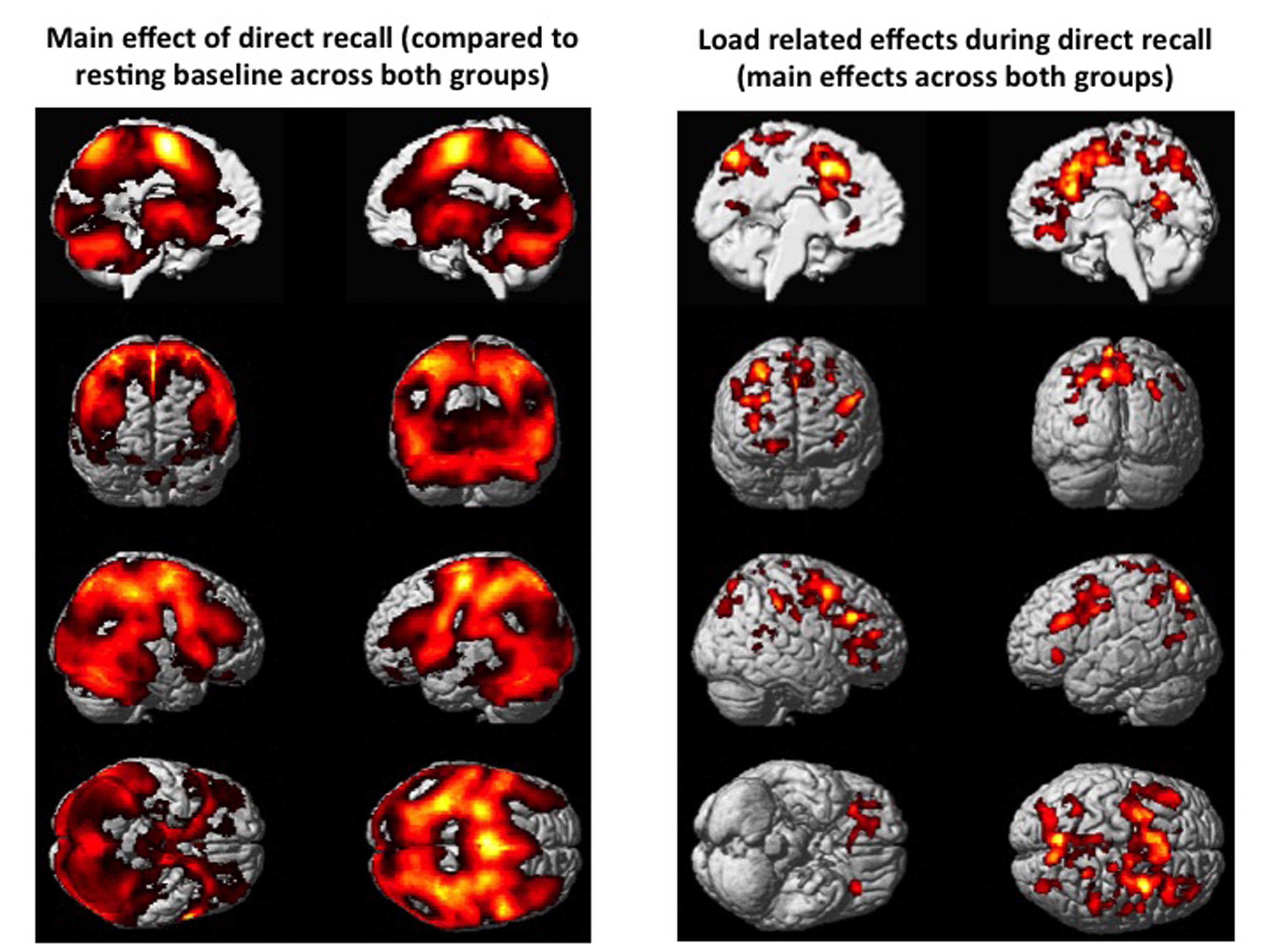

Supplement: Figure S2 — Left side - main effect of direct recall (compared to resting baseline across both groups). Right side - load related effects during direct recall (main effects across both groups). (TIF) [file pone.0061786.s002.tif]

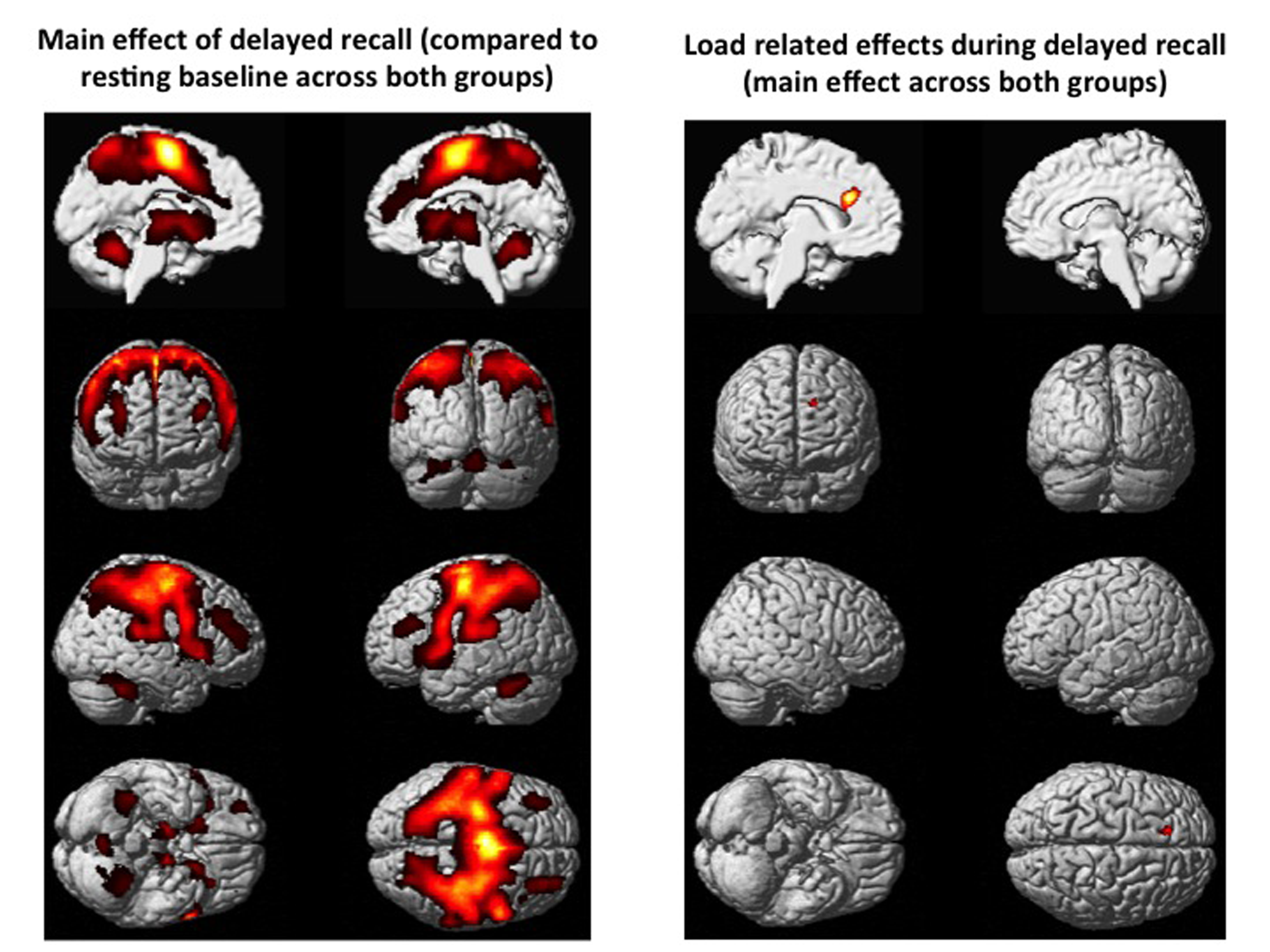

Supplement: Figure S3 — Left side - main effect of delayed recall (compared to resting baseline across both groups). Right side - load related effects during delayed recall (main effects across both groups). (TIF) [file pone.0061786.s003.tif]

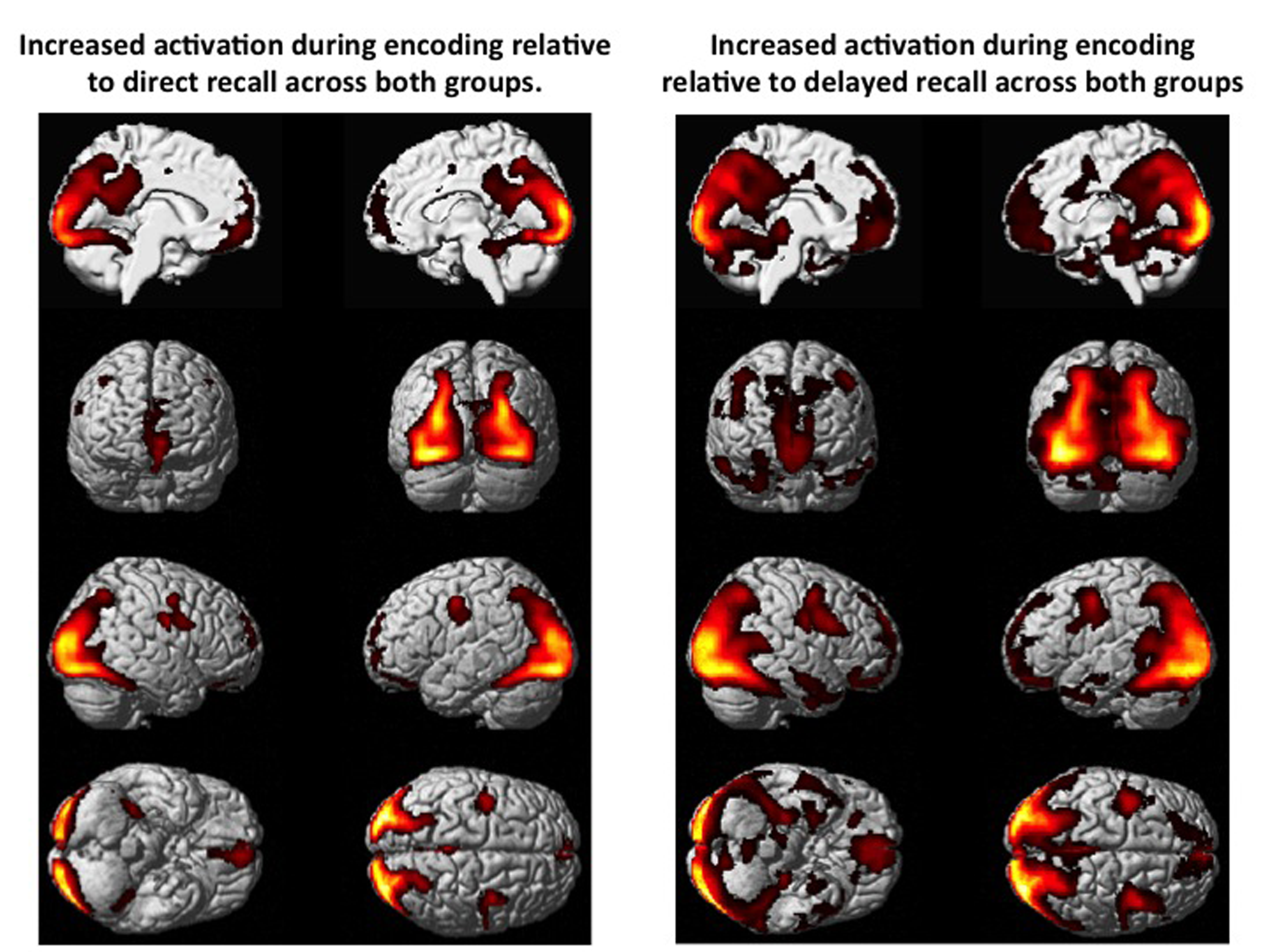

Supplement: Figure S4 — Left side – increased activation during encoding relative to direct recall across both groups. Right side - increased activation during encoding relative to delayed recall across both groups. (TIF) [file pone.0061786.s004.tif]

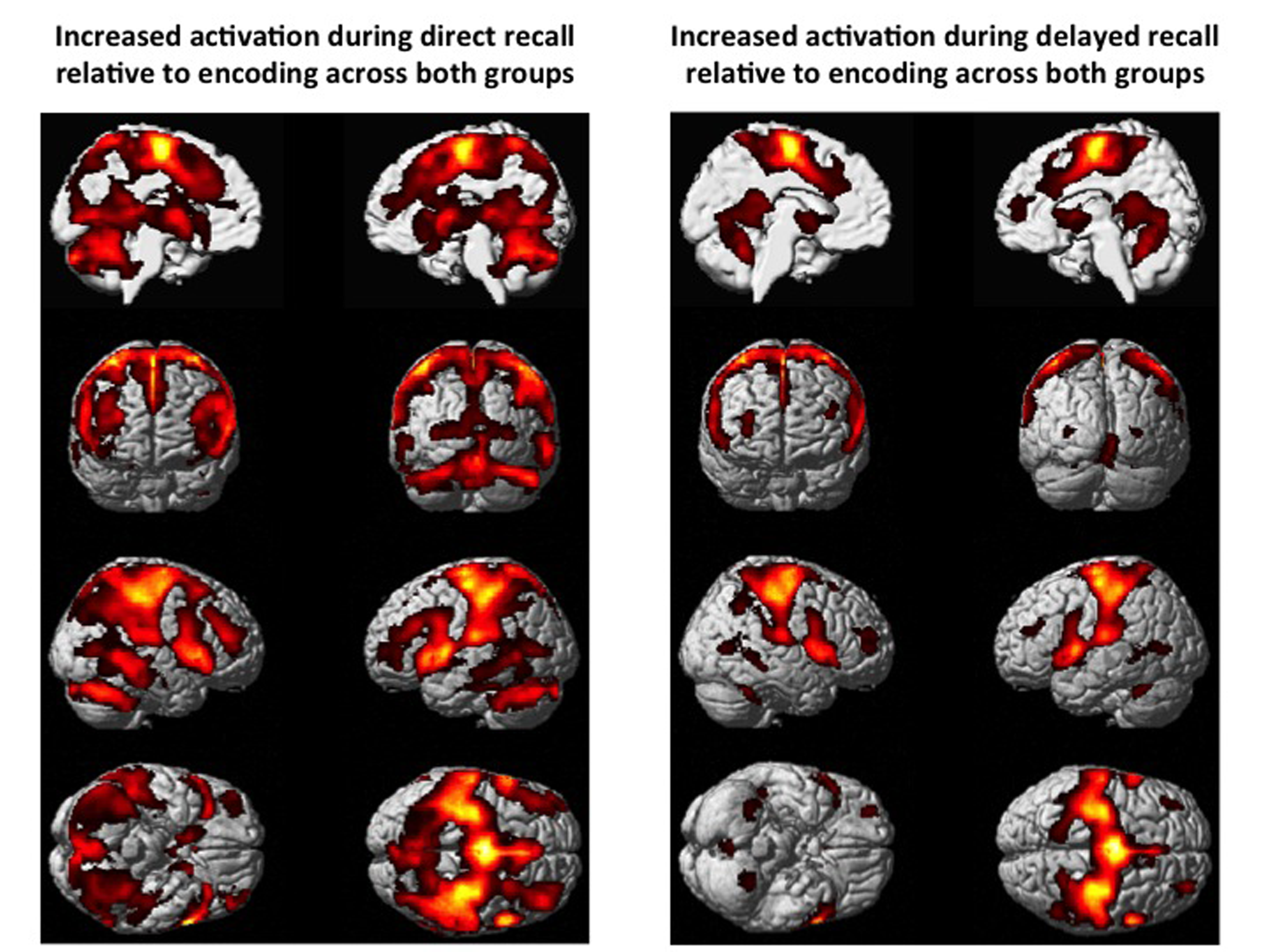

Supplement: Figure S5 — Left side – increased activation during direct recall relative to encoding across both groups. Right side - increased activation during delayed recall relative to encoding across both groups. (TIF) [file pone.0061786.s005.tif]

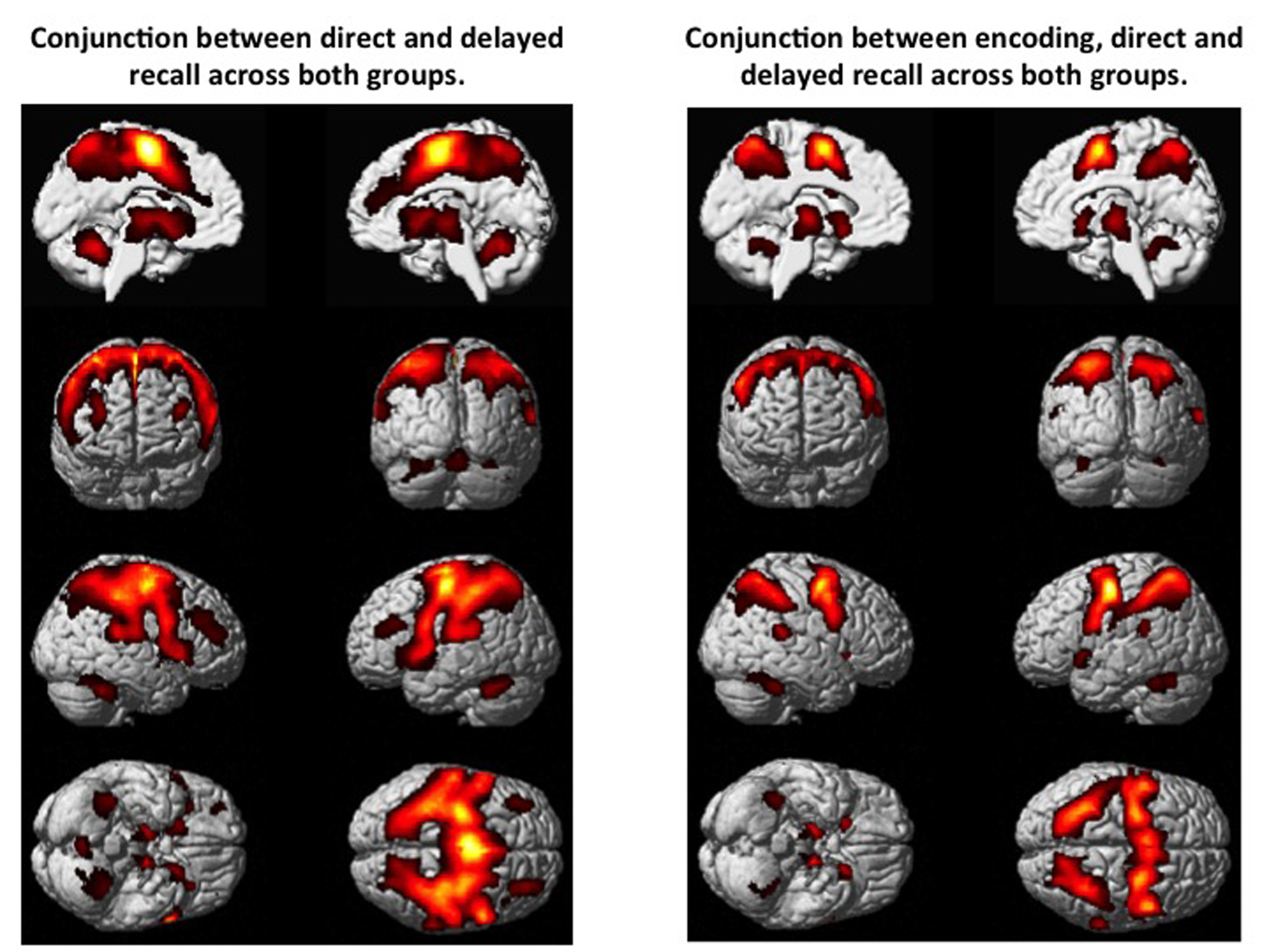

Supplement: Figure S6 — Left side – conjunction between direct and delayed recall across both groups. Right side - conjunction between encoding, direct and delayed recall across both groups. (TIF) [file pone.0061786.s006.tif]

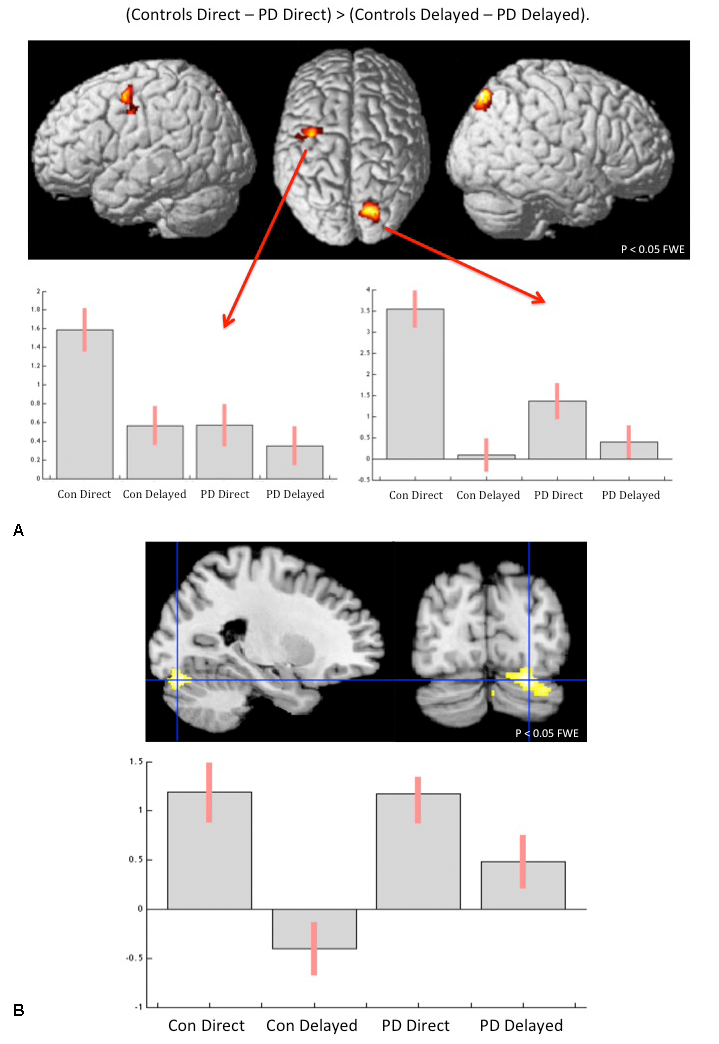

Supplement: Figure S7 — A - Interaction (ConDirect – PatDirect)>(ConDelayed – PatDelayed): Regions in which patients showed a significant specific reduction of activity during direct retrieval as tested by the interaction (ConDirect – PatDirect)>(ConDelayed – PatDelayed) in conjunction with the respective main effect (ConDirect – PatDirect), as well as the mean parameter estimates and 90% confidence intervals for the individual conditions at the location of the local maxima. B - Interaction (PatDelayed – ConDelayed)>(PatDirect – ConDirect): Regions in which patients showed a significant specific increase of activity during delayed retrieval as tested by the interaction (PatDelayed – ConDelayed)>(PatDirect – ConDirect) in conjunction with the respective main effect (PatDelayed – ConDelayed), as well as the mean parameter estimates and 90% confidence intervals for the individual conditions at the location of the local maxima. (TIF) [file pone.0061786.s007.tif]
