# Supplementary material for: Diminished Activation of Motor Working-Memory Networks in Parkinson's Disease
Source: PLoS One. 2013 Apr 19;8(4):e61786. doi: 10.1371/journal.pone.0061786 (PMC3631252; doi:10.1371/journal.pone.0061786)
Supplement: Table S1 — Working memory task performance accuracy in patients with Parkinson's disease (PD) and healthy controls (HC) during direct recall, delayed recall and all conditions. Hits and misses are given for the 4-sequence and 5-sequence. (DOC) [file pone.0061786.s008.doc]

S-Table 1 – Working memory task performance accuracy

|  |  |  | ***PD*** | ***HC*** |
| --- | --- | --- | --- | --- |
|  |  |  |  |  |
| Direct recall | 4-sequence | Hits | 5.09 ± 3.27 | 8.57 ± 2.48 |
|  |  | Misses | 6.91 ± 3.27 | 3.43 ± 2.48 |
|  | 5-sequence | Hits | 4.04 ± 3.11 | 5.83 ± 3.04 |
|  |  | Misses | 7.96 ± 3.11 | 6.17 ± 3.04 |
| Delayed recall | 4-sequence | Hits | 4.74 ± 3.49 | 7.77 ± 2.84 |
|  |  | Misses | 7.26 ± 3.49 | 4.23 ± 2.84 |
|  | 5-sequence | Hits | 2.48 ± 2.91 | 5.08 ± 3.08 |
|  |  | Misses | 9.52 ± 1.91 | 6.60 ± 3.32 |
| All conditions |  | Hits | 4.09 ± 3.31 | 6.78 ± 3.17 |
|  |  | Misses | 7.91 ± 3.31 | 5.15 ± 3.19 |
|  |  |  |  |  |
